# Temporal Trends and Disparities in Hypertension‐Related Cardiomyopathy Mortality in the United States, 1999–2023

**DOI:** 10.1002/clc.70417

**Published:** 2026-07-17

**Authors:** Javeria Akhter, Iqra Taj, Muhammad Umar, Abdulrahman Nasir Al Khatib, Jamil Nasrallah

**Affiliations:** ^1^ Lung Health Program, Community Health Directorate Indus Hospital and Health Network Karachi Pakistan; ^2^ Jinnah Sindh Medical University Karachi Pakistan; ^3^ Khairpur Medical College Khairpur Pakistan; ^4^ School of Medicine, University of Jordan Amman Jordan; ^5^ Department of Medicine Lebanese University Beirut Lebanon

**Keywords:** cardiomyopathy, CDC wonder, hypertension, mortality, United States

## Abstract

**Background:**

Hypertension and cardiomyopathy are major contributors of cardiovascular morbidity in the United States. This study investigates long‐term mortality trends and disparities in hypertension‐related cardiomyopathy from 1999 to 2023 in the United States.

**Methods:**

Mortality data from CDC WONDER (1999–2023) were evaluated for cardiomyopathy (ICD‐10 I42, I42.2, and I42.9) as the primary cause of death and hypertension (ICD‐10 I10, I15.0 and I15.9) as a contributing cause among individuals aged ≥ 25 years. Age‐adjusted mortality rates (AAMRs) were standardized to the United States 2000 population. Joinpoint regression estimated annual percent change (APC) with 95% confidence intervals.

**Results:**

Between 1999 and 2023, 153 563 deaths were identified. AAMR increased from 1.26 per 100 000 in 1999 to 2.22 in 2023, driven by a significant early rise from 1999 to 2001 (APC: +30.33; *p* < 0.005). Males had higher mortality than females (2.98 vs. 1.61) with males declining significantly from 2001 to 2023 (APC: −0.64; *p* < 0.005). NH Black individuals had the highest AAMR (10.81), followed by NH White individuals (5.02). Mortality was higher in non‐metropolitan than metropolitan areas (2.71 vs. 2.26), both declining significantly after 2001 (APC: −0.89; −1.69; *p* < 0.005). South had the highest burden (AAMR: 4.86), while the Midwest rose significantly after 2017 (APC: +4.11; *p* < 0.005). Adults aged ≥ 85 years had the highest mortality (CMR: 32.79) increasing significantly after 2015 (APC: +2.44 *p* < 0.005).

**Conclusion:**

Hypertension‐related cardiomyopathy mortality rose sharply in 1999–2001 before declining through 2023, with persistent disparities by sex, race, geography, and age. Targeted hypertension control and equitable cardiovascular care remain needed to reduce this burden.

## Graphical abstract

Hypertension‐related cardiomyopathy mortality in the United States increased sharply in 1999–2001 before declining through 2023. Persistent disparities by sex, race, geography, and age highlight the need for improved hypertension control, early cardiomyopathy management, and equitable access to cardiovascular care.

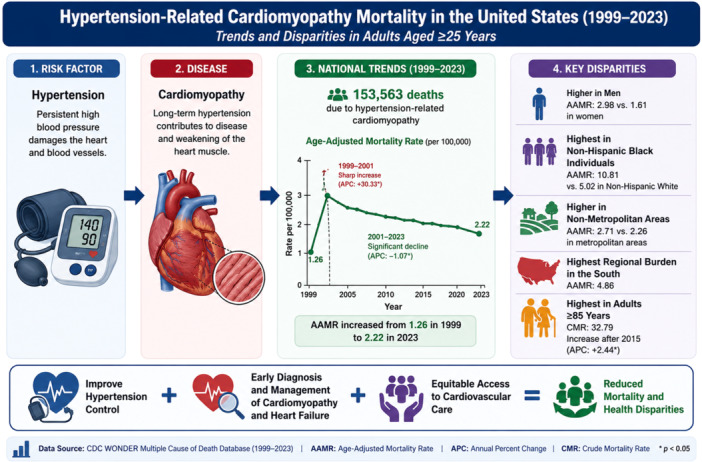

## 1 Introduction

Hypertension and cardiomyopathy are two important factors that substantially contribute to cardiovascular disease and death in the United States (US). Hypertension, which is defined as consistently raised arterial blood pressure, affects nearly 50% of the adult population [1]. Recent data indicate that approximately 122 million (47.3%) of all adults in the US have hypertension. Although there are effective antihypertensive treatments exists, controlling blood pressure adequately remains a significant public health challenge, predominantly among Black individuals and older adults [2]. Long term uncontrolled blood pressure is a recognized risk factor for structural heart changes, such as left ventricular hypertrophy and fibrosis, which can result in hypertensive cardiomyopathy and heart failure [3].

Cardiomyopathies include a varied group of disorders affecting the cardiac muscle, which may stem from genetic mutations, metabolic disorders, decreased blood flow (ischemia), or long‐term stress on the heart caused by conditions like hypertension. Hypertensive heart disease is among most common forms of secondary cardiomyopathy in the United States [4]. Beyond its direct health effect, cardiomyopathy is a leading cause of sudden cardiac death and hospitalizations due to heart failure and its complex association with systemic vascular disease is increasingly recognized [5].

Mechanistically, chronic hypertension increases cardiac afterload, promoting left ventricular hypertrophy, myocardial fibrosis, and diastolic dysfunction; over time, this remodeling can progress to overt hypertensive cardiomyopathy and heart failure [6]. In this causal sequence, hypertension acts as the principal upstream risk factor, whereas the resulting cardiomyopathy—through progressive pump failure, arrhythmia, or sudden cardiac death—represents the proximate cause of death typically recorded on the death certificate.

Prior CDC WONDER‐based analyses have characterized mortality trends for hypertrophic cardiomyopathy, generally reporting an overall decline in age‐adjusted mortality alongside persistent disparities by sex, race, and age. However, these prior studies have not evaluated hypertension as a contributing cause of death, nor have they tracked cardiomyopathy‐related mortality specifically linked to hypertension across the full 1999–2023 period [7]. Understanding these variations is crucial to refining public health interventions, optimizing cardiovascular risk reduction strategies, and decreasing preventable deaths.

Given the well‐established pathophysiological link between hypertension and cardiomyopathy, the persistent demographic and geographic disparities in cardiovascular mortality, and the lack of a comprehensive national analysis focused specifically on this cause‐of‐death combination, a dedicated evaluation of long‐term trends in hypertension‐related cardiomyopathy mortality is warranted. Therefore, in this study, data from the Centers for Disease Control and Prevention's Wide‐ranging Online Data for Epidemiologic Research (CDC WONDER) platform are utilized to analyze population‐based trends in hypertension and cardiomyopathy‐related mortality in the United States from 1999 to 2023. By examining demographic disparities and temporal changes in cause‐specific mortality, this research seeks to guide targeted prevention efforts, enhance early detection and management of hypertensive heart disease, and provide valuable insights for clinicians and policymakers working to mitigate this persistent public health burden.

## 2 Methods

### 2.1 Study Setting and Population

A retrospective cohort study was performed to evaluate the cardiomyopathy‐ related mortality rate in patients with hypertension as a comorbidity in the United States from 1999 to 2023. Mortality data were obtained from the Centers for Disease Control and Prevention's WONDER (Wide‐Ranging Online Data for Epidemiologic Research) database among hypertensive patients (age > 25) who died of cardiomyopathy. Our focus was explicitly on Multiple Cause‐of‐Death (MCD = 10) Public Use Record database to identify cases where cardiomyopathy was recorded as the underlying cause and hypertension as the contributing cause of mortality [8]. Individuals were identified using the International Classification of Diseases, 10th Revision, Clinical Modification (ICD‐10‐CM) codes: I10 (essential (primary) hypertension), I15.0 (renovascular hypertension), and I15.9 (secondary hypertension, unspecified) for hypertension, as well as I42.0 (dilated cardiomyopathy), I42.2 (other hypertrophic cardiomyopathy), and I42.9 (cardiomyopathy, unspecified) for cardiomyopathy, specifically for individuals aged 25 years and older. The same codes were previously used to identify mortality related to hypertension and cardiomyopathy in administrative data [9, 10]. Mortality data derived from death certificates may be subject to misclassification of underlying and contributing causes of death. Institutional review board (IRB) approval was not required because the dataset is publicly available and fully de‐identified. Strengthening the Reporting of Observational Studies in Epidemiology (STROBE) guidelines was used in this study [11].

### 2.2 Data Abstraction

We extracted data of individuals age > 25 on mortality related to hypertension and cardiomyopathy, including population estimates, demographics (age, sex, and race/ethnicity), and geographic information (state and urban‐rural classification) for the period from 1999 to 2023. Race and ethnicity were categorized using bridged‐race categories, including non‐Hispanic (NH) White, NH Black or African American, NH Other (which encompasses NH Asian or Pacific Islander, NH American Indian or Alaska Native), and Hispanic or Latino provided by the National Center for Health Statistics (NCHS), which standardize racial/ethnic classifications across changes in US Census definitions over time to ensure temporal comparability. Age was divided into the following groups: 25–34, 35–44, 45–54, 55–64, 65–74, 75–84, and 85 years and older. We also analyzed mortality trends across individual states, US Census regions (Northeast, Midwest, South, and West), and by urban‐rural status at the county level. National Center for Health Statistics Urban‐Rural Classification Scheme 2013 was used to classified counties as rural (micropolitan and noncore areas) or urban (large central metro, large fringe metro, medium metro, and small metro) [12].

### 2.3 Statistical Analysis

Crude mortality rates (CMRs) and age‐adjusted mortality rates (AAMRs) per 100 000 population were calculated. CMRs were determined by dividing the number of deaths attributed to hypertension and cardiomyopathy each year by the corresponding US population. The US population as of the year 2000 was used as the standard for AAMR calculations [13]. To evaluate mortality pattern in hypertension‐related cardiomyopathy the Joinpoint Regression Program (Joinpoint version 5.2.0) was utilized [14]. This program identifies significant changes in annual mortality trends over time through Joinpoint regression, fitting linear segments where noteworthy temporal differences occurred. Monte Carlo permutation test was used to calculated Annual percentage change (APC) and 95% confidence intervals (CIs) from AAMRs and standard error. To capture the overall mortality trend for the study period, the weighted average of the APCs was summarized along with their corresponding 95% CIs. An APC was considered to be increasing or decreasing if the slope describing the change in mortality was significantly different from 0 based on 2‐tailed *t*‐testing. A *p* value of less than 0.05 was considered statistically significant. Additionally, we conducted a sensitivity analysis that focused on deaths where hypertension was listed as the underlying cause and cardiomyopathy as a contributing cause, and vice versa. A pairwise Joinpoint comparison was also performed to test whether the two series exhibited parallel or coincident trends. Statistical significance was set at *p* < 0.05 [15].

## 3 Results

A total of 153 563 hypertension‐related cardiomyopathy deaths occurred in individuals aged ≥ 25 years between 1999 and 2023. Among these deaths, 33.57% occurred in Decedent's home, 28.56% occurred in in‐patients and 17.72% occurred in nursing homes (Supporting Information S1: Table 1).

### 3.1 Annual Trends in AAMRs for Cardiomyopathy Related to Hypertension

**Figure 1:**
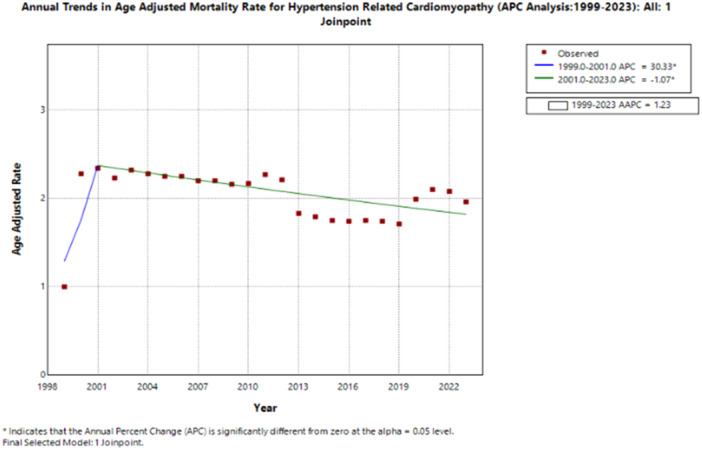
Overall age‐adjusted mortality rate for hypertension related cardiomyopathy in the United States, 1999−2023.

For individuals aged 25 years and older, the AAMR for cardiomyopathy deaths related to hypertension was 1.26 in 1999 and increased to 2.22 in 2023. From 1999 to 2001, the AAMR showed a significant increase (APC: 30.33; 95% CI: 4.36 to 62.75) and from 2001 to 2023, the AAMR showed a significant decline (APC: −1.07; 95% CI: −1.54 to −0.58) (Supporting Information S1: Table 2) (Figure 1).

### 3.2 Cardiomyopathy Related to Hypertension AAMR Stratifies by Sex

**Figure 2:**
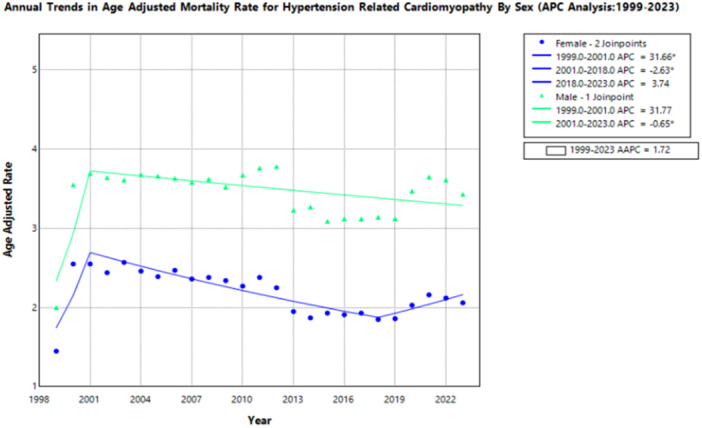
Trends in age‐adjusted mortality rates for hypertension related cardiomyopathy by sex in the United States, 1999–2023.

Throughout the study period, males consistently exhibited higher AAMRs than females. Among males, AAMRs increased significantly during the early period (1999–2001) (APC: +31.77%; 95% CI: −0.74 to 74.93), followed by a statistically significant decline from 2001 to 2023 (APC: −0.64%; 95% CI: −1.15 to −0.13), resulting in a decrease from 3.24 in 2001 to 2.98 in 2023. Comparably, female AAMRs showed a significant increase during 1999–2001 (APC: +31.66%; 95% CI: 3.53 to 67.43), followed by a significant decline from 2001 to 2018 (APC: −2.63%; 95% CI: −3.34 to −1.97). This was followed by a non‐significant upward trend from 2018 to 2023 (APC: +3.73%; 95% CI: −0.51 to 8.18), with rates increasing from 1.40 in 2018 to 1.61 in 2023 (Figure 2).

### 3.3 AAMRs for Hypertension‐Related Cardiomyopathy, Stratified by Geographic Region

At the state level, marked variation in AAMRs was observed. Massachusetts had one of the lowest AAMRs (2.08; 95% CI: 1.89–2.27), whereas Delaware recorded one of the highest AAMRs (8.38; 95% CI: 7.33–9.44) (Supporting Information S1: Table 4).

**Figure 3:**
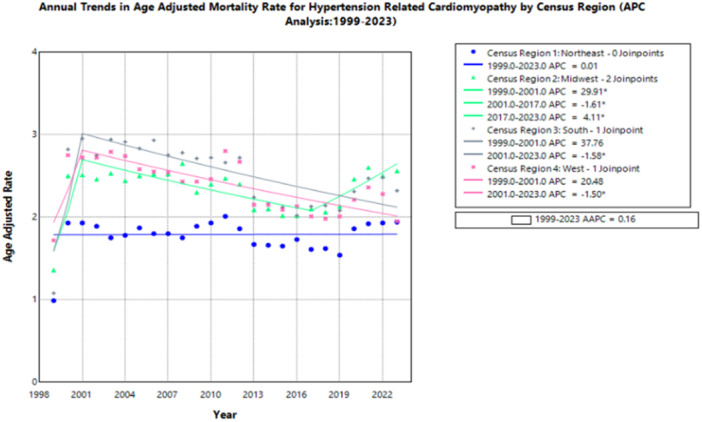
Trends in age‐adjusted mortality rates for hypertension related cardiomyopathy by census region in the United States, 1999−2023.

At the Census region level, the Southern region demonstrated the highest overall mortality burden (AAMR: 4.86; 95% CI: 4.79–4.93), with a significant declining trend observed over the study period (2001–2023) (APC: –1.58; 95% CI: –2.18 to –0.98). The Midwestern region showed a similar overall burden (AAMR: 4.84; 95% CI: 4.75–4.94), with a significant decline from 2001 to 2017 (APC: –1.61; 95% CI: –2.36 to –0.85), followed by a significant reversal with increasing mortality from 2017 to 2023 (APC: +4.11; 95% CI: 1.23 to 7.07). The Western region demonstrated a consistently lower burden compared with the South and Midwest and showed a sustained significant decline across the study period (2001–2023) (APC: –1.53; 95% CI: –2.04 to –0.96) (Figure 3; Supporting Information S1: Table 5).

### 3.4 AAMRs for Hypertension‐Related Cardiomyopathy, Stratified by Urbanization

Throughout the study period, non‐metropolitan areas consistently exhibited higher AAMRs compared with metropolitan areas (2.71; 95% CI: 2.68–2.75 vs. 2.26; 95% CI: 2.25–2.28, respectively).

**Figure 4:**
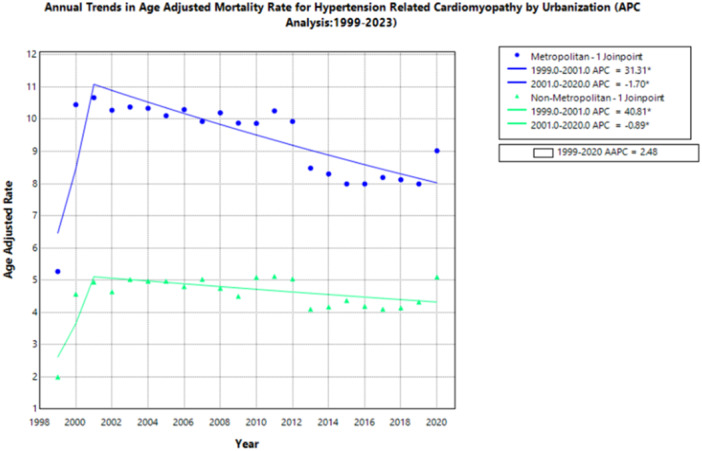
Trends in age‐adjusted mortality rates for hypertension related cardiomyopathy by urbanization in the United States, 1999‐2020.

In non‐metropolitan areas, AAMRs increased significantly during the early study period (1999–2001) (APC: +40.80%; 95% CI: 2.33–93.74), followed by a statistically significant decline from 2001 to 2023 (APC: –0.89%; 95% CI: –1.57 to –0.20). Similarly, metropolitan areas demonstrated a significant increase during 1999–2001 (APC: +31.31%; 95% CI: 3.98–65.80), followed by a significant decline from 2001 to 2023 (APC: –1.69%; 95% CI: –2.23 to –1.17), although the rate of decline was steeper compared with non‐metropolitan areas (Figure 4) (Supporting Information S1: Table 6).

### 3.5 AAMRs for Hypertension‐Related Cardiomyopathy, Stratified by Race/Ethnicity

When stratified by race/ethnicity, non‐Hispanic (NH) Black or African American individuals exhibited the highest AAMRs, followed by NH White, American Indian or Alaska Native, Hispanic or Latino, and NH Asian or Pacific Islander populations. The overall AAMRs were as follows: NH Black or African American: 10.81 (95% CI: 10.59–11.04); NH White: 5.02 (95% CI: 4.98–5.08); American Indian or Alaska Native: 4.59 (95% CI: 4.07–5.15); Hispanic or Latino: 4.18 (95% CI: 4.03–4.32); and NH Asian or Pacific Islander: 2.98 (95% CI: 2.81–3.16).

Over the study period, NH Black or African American individuals showed a non‐significant change in AAMRs from 1999 to 2009 (APC: +33.07%; 95% CI: –2.37 to 81.41), followed by a significant declining trend from 2009 to 2017 (APC: –3.87%; 95% CI: –4.93 to –2.80).

NH Asian or Pacific Islander individuals demonstrated a significant declining trend from 1999 to 2023 (APC: –3.67%; 95% CI: –4.65 to –2.68), with a continued but non‐significant decline from 2021 to 2023 (APC: –9.80%; 95% CI: –65.44 to 135.41).

**Figure 5:**
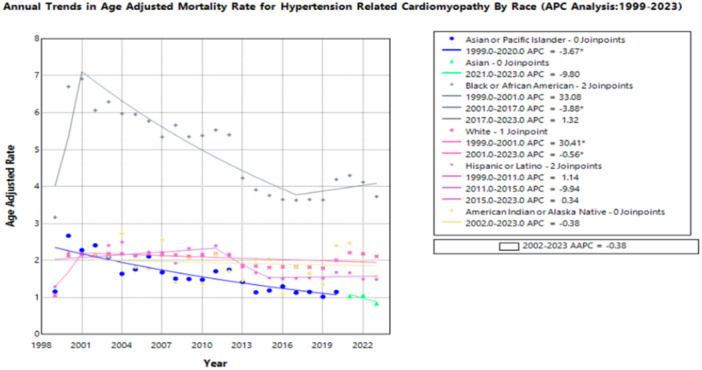
Trends in age‐adjusted mortality rates for hypertension related cardiomyopathy in the United States by Race, 1999–2023.

NH White individuals experienced a significant increase during the early period (1999–2001) (APC: +30.41%; 95% CI: 2.53–65.87), followed by a sustained significant decline from 2001 to 2023 (APC: –0.56%; 95% CI: –1.04 to –0.07). No statistically significant temporal trends were observed among Hispanic or Latino and American Indian or Alaska Native populations over the study period, indicating overall stability in mortality trends (Figure 5) (Supporting Information S1: Table 6).

### 3.6 AAMRs for Hypertension‐Related Cardiomyopathy, Stratified by 10‐Year Age Group

When stratified by ten‐year age groups, crude mortality rates (CMRs) were highest among individuals aged 85 years and older, followed by those aged 75–84, 65–74, 55–64, 45–54, and 35–44 years (CMR: 32.79; 95% CI: 31.97–33.61; 11.31; 95% CI: 11.02–11.60; 5.10; 95% CI: 4.96–5.24; 2.53; 95% CI: 2.44–2.62; 1.13; 95% CI: 1.07–1.19; and 0.44; 95% CI: 0.41–0.48, respectively).

Among individuals aged 85 years and older, CMRs showed a significant increase between 1999 and 2001 (APC: +39.61%; 95% CI: 18.27–64.79), followed by a period of stability from 2001 to 2011. This was followed by a significant decline between 2011 and 2015 (APC: –8.74%; 95% CI: –13.73 to –3.47), and a subsequent significant increase from 2015 to 2023 (APC: +2.44%; 95% CI: 1.19–3.71), reaching 32.8 in 2023.

In the 65–74‐year age group, CMRs increased significantly between 1999 and 2001 (APC: +26.79%; 95% CI: 0.07–60.63), followed by a sustained decline from 2001 to 2016 (APC: –2.49%; 95% CI: –3.41 to –1.57), and a subsequent increase from 2016 to 2023 (APC: +2.86%; 95% CI: 0.46–5.32).

The 75–84‐year age group demonstrated a continuous and significant decline over the study period (APC: –1.13%; 95% CI: –1.91 to –0.35).

**Figure 6:**
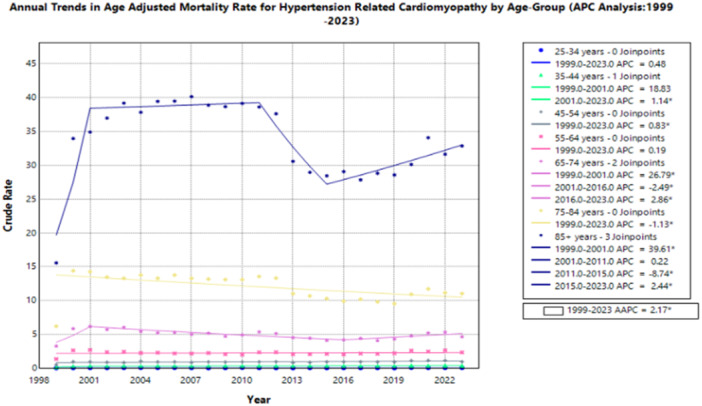
Trends in crude mortality rate for hypertension related cardiomyopathy in the United States by Age‐Group, 1999–2023.

For younger age groups, CMRs showed smaller but statistically significant increases over time in individuals aged 35–44 years (2001–2023; APC: +1.14%; 95% CI: 0.55–1.73) and 45–54 years (full study period; APC: +0.83%; 95% CI: 0.23–1.43), while no statistically significant temporal trends were observed in the 25–34 and 55–64‐year age groups (Figure 6) (Supporting Information S1: Table 7).

### 3.7 Sensitivity Analysis and Pairwise Comparison

**Figure 7:**
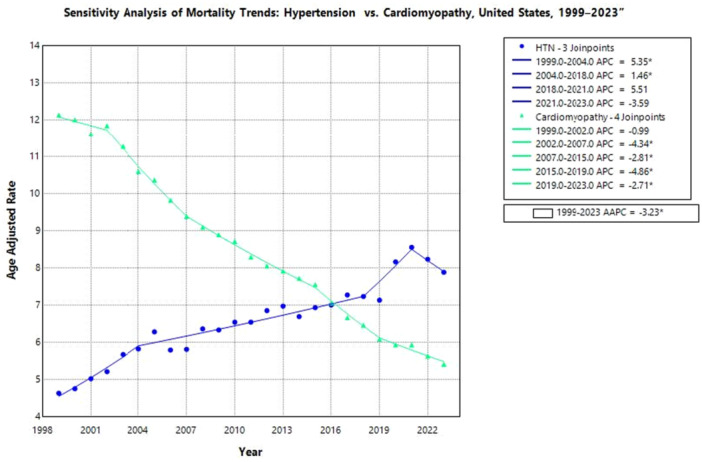
Sensitivity analysis of mortality trends: hypertension and cardiomyopathy.

The AAMR for hypertension increased from 4.6 per 100 000 in 1999 to 8.0 in 2023. Joinpoint regression identified three significant inflection points. The most rapid increase occurred during 1999–2004 (APC = +5.35%, *p* < 0.05) and 2018–2021 (APC = +5.51%, *p* < 0.05), followed by a modest decline after 2021 (APC = −3.59%). In comparison, cardiomyopathy mortality declined steadily from 12.1 to 6.3 per 100 000 during the same period, with four Joinpoint identified. The steepest declines were observed between 2002 and 2007 (APC = −4.34%, *p* < 0.05) and 2015–2019 (APC = −4.86%, *p* < 0.05), yielding an overall AAPC of −3.23% (*p* < 0.05) (Figure 7).

**Figure 8:**
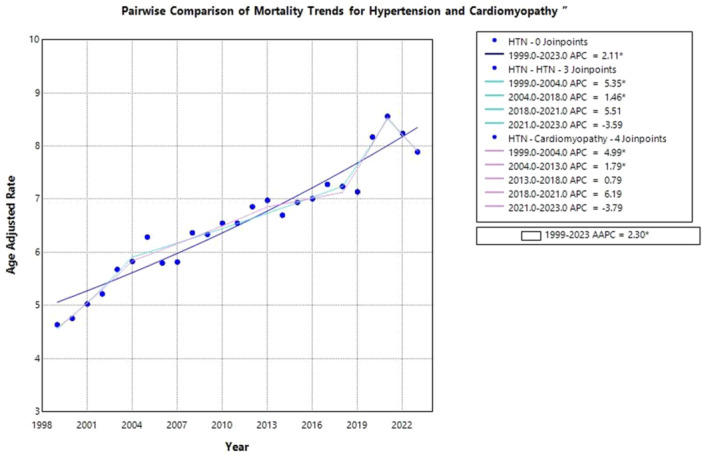
Pairwise joinpoint comparison: hypertension and cardiomyopathy.

The pairwise comparison test was significant (*p* < 0.05), indicating non‐parallel trends between hypertension and cardiomyopathy mortality (Figure 8).

## 4 Discussion

A 25‐year mortality analysis using data from the CDC WONDER database highlighted significant trends and disparities in hypertension‐related cardiomyopathy deaths in the United States. Although mortality increased significantly between 1999 and 2001, rates subsequently declined from 2001 to 2023. Mortality burden remained disproportionately high among males, non‐Hispanic Black individuals, residents of non‐metropolitan areas, and older adults, particularly those aged ≥ 85 years. Regional differences were also observed, with the South showing the highest mortality burden and the Midwest demonstrating a recent increase after 2017.

A large number of deaths occurred either at home or in nursing facilities rather than in hospitals, suggesting a significant gap in the identification and treatment of hypertension and its chronic cardiac sequelae within community settings.

Although the overall AAMR for hypertension‐related cardiomyopathy increased significantly from 1999 to 2001, it subsequently decreased through 2023. The pronounced early increase is likely attributable to methodological and coding transitions during the early ICD‐10 implementation period, improved completeness of MCD reporting, and low baseline counts in rare cause‐specific mortality categories, which may have amplified early percentage changes [16]. The subsequent decline likely reflects a combination of evolving diagnostic criteria and treatment modalities: refinement of ICD‐10‐CM subclassification of cardiomyopathy phenotypes and wider adoption of echocardiographic and natriuretic peptide‐based diagnostic algorithms improved case ascertainment over time, while expanded use of guideline‐directed therapies—including RAAS inhibitors, beta‐blockers, SGLT2 inhibitors, and device‐based interventions—alongside enhanced population‐level detection and management of hypertension and cardiovascular risk factors, likely contributed to the sustained decline in mortality after the early study period [17, 18, 19, 20].

Sex‐stratified analysis confirmed that males consistently exhibited higher AAMRs than females, aligning with prior literature on sex‐specific left ventricular remodeling and fibrosis and hypertension burden among males [21, 22]. Regarding metabolic risk clustering, NHANES‐based analyses showed that younger men most frequently exhibit the triad of high triglycerides, low HDL and elevated BP, consistent with a more atherogenic metabolic pattern, while component trends (e.g., central adiposity and low HDL) have increased in both sexes [23]. In comparison, females demonstrated lower mortality rates overall, which may reflect differences in cardiovascular risk profiles and greater utilization of preventive healthcare services [24]. However, increasing hypertension prevalence and heart‐failure risk with aging may partly explain the less favorable mortality risk with aging may partly explain the less favorable trends observed in older women [25].

The geographic differences were remarkable. The South demonstrated the highest mortality burden, while the Midwest showed a concerning increase after 2017. These differences are probably due to regional variations in the prevalence of hypertension, obesity, diabetes, and access to healthcare, as well as broader environmental and healthcare‐system factors [26, 27]. Previous studies have similarly reported elevated cardiovascular mortality in southern and midwestern states, partly attributable to higher cardiometabolic risk factors and limited access to specialty cardiovascular care [28, 29, 30, 31].

The disparities between urban and rural were also identified, with non‐metropolitan areas reported higher AAMRs, a pattern that is consistent with the well‐established pattern of increased cardiovascular mortality in rural areas. The reason for the increase in AAMR is due to the poorer cardiovascular outcomes in rural populations, potentially related to reduced healthcare access, delayed diagnosis, lower availability of specialty services, and socioeconomic disparities [32, 33, 34].

One of the most pronounced findings in this analysis was the racial and ethnic disparities. NH Black African populations experienced the highest mortality, consistent with well‐documented inequities in hypertension prevalence, earlier onset, resistant hypertension, and lower control rates [35, 36]. These differences are deeply rooted in structural determinants of health—including segregation, income/wealth inequities, underinsurance, distance to specialty care, adverse food and environmental exposures—accumulate over the life course to worsen cardiomyopathy risk and outcomes [37, 38]. Among American Indian/Alaskan Native communities, chronic underfunding, geographic barriers, and persistent infrastructure limitations within and around Indian Health Service (HIS) facilities which may contribute to poorer cardiomyopathy outcomes [39].

The age‐group analysis exhibited that older adults, especially those aged > 85 years, had the highest mortality rates, with rates increased noticeably after 2015. This finding is consistent with prior studies identifying older age as a significant prognosticator of poor outcomes in cardiomyopathy, reflecting the combined effects of long‐term uncontrolled blood pressure, microvascular dysfunction, and multimorbidity including CKD, diabetes and atrial fibrillation [40, 41].

The increase after 2015 may reflect: (i) an increase in the aging population (5.1−5.7 million); (ii) a shift toward HFpEF (more prevalent in older adults with hypertension and obesity); (iii) polypharmacy and physical weakness which restrict the up‐ titration of guideline‐directed therapies; and (iv) coding granularity after ICD‐10 adoption [42]. In contrast, younger adults displayed a relatively constant trend, consistent with increased treatment response and cardiac reverse remodeling under current antihypertensive therapy. Notably, evidence indicates that age alone should not prevent aggressive risk‐factor modification or cardiomyopathy treatment. Older adults can benefit from RAAS blockade, SGLT2 inhibitors for appropriate phenotypes, and multidisciplinary HF care, which remain the cornerstone of evidence‐based therapies [43].

These findings have important public health and clinical implications. Persistent disparities across sex, race, age, and geographic regions highlight the need for targeted hypertension screening, improved blood pressure control, and equitable access to evidence‐based cardiovascular care, particularly in high‐risk populations. Strengthening preventive cardiology services and expanding healthcare access in underserved communities may help reduce the burden of hypertension‐related cardiomyopathy mortality in the United States.

### 4.1 Limitations

Limitations of the study should be recognized. Firstly, because the study relies on ICD codes and death certificates, there is a possibility for data misrepresentation and omissions, which could lead to imprecision or underestimation of true mortality rates. Secondly, the ecological design limited adjustment for individual‐level factors such as comorbidities, treatment adherence, and socioeconomic status. The data set did not include the details of the clinical parameters and the treatment and management of the patients. Additionally, reliance on death certificate data introduces potential variability in physician reporting practices across jurisdictions, which may influence geographic comparisons. Lastly, these findings cannot prove cause and effect at the individual level, although they demonstrate population‐level associations.

## 5 Conclusion

Hypertension‐related cardiomyopathy mortality in the United States rose sharply in the early study period before declining, with the highest burden among males, NH Black individuals, older adults, and residents of southern and non‐metropolitan regions. These findings underscore the need for targeted hypertension prevention, equitable cardiovascular care, and implementation of evidence‐based management strategies to reduce mortality in high‐risk populations.

## Author Contributions

Conceptualization: Javeria Akhter. Methodology: Javeria Akhter. Data curation: Javeria Akhter. Formal analysis: Javeria Akhter. Writing–original draft: Muhammad Umar, Iqra Taj, Abdulrahman Nasir Al Khatib. Writing–review and editing: Javeria Akhter, Jamil Nasrallah. Supervision: Jamil Nasrallah. All authors approved the final version of the manuscript.

## Funding

The authors have nothing to report.

## Ethics Statement

Due to the de‐identified nature, the need for Institutional Review Board approval is waived.

## Consent

Due to the de‐identified nature, the need for consent to participate is waived. The final draft of this work has been read and approved by all authors, who also consent to its publication.

## Conflicts of Interest

The authors declare no conflicts of interest.

## Supporting information


Supporting File

## Data Availability

All data utilized in this analysis are publicly available and have been sourced from publicly accessible CDC WONDER database.
