# Supplementary material for: Temporal Trends and Disparities in Hypertension‐Related Cardiomyopathy Mortality in the United States, 1999–2023
Source: Clin Cardiol. 2026 Jul 17;49(7):e70417. doi: 10.1002/clc.70417 (PMC13377619; doi:10.1002/clc.70417)
Supplement: Supplementary file 1 — Supporting File [file CLC-49-e70417-s001.docx]

**Supplementary Material**

**Supplementary Table 1.** Place of Death for Hypertension-Related mortality trends in Cardiomyopathy in the United States, 1999-2023

| Place of Death | Deaths | % of Total Deaths |
| --- | --- | --- |
| Medical Facility - Inpatient | 43857 | 28.56% |
| Medical Facility - Outpatient or ER | 17180 | 11.19% |
| Medical Facility - Dead on Arrival | 1286 | 0.84% |
| Medical Facility - Status unknown | 111 | 0.07% |
| Decedent's home | 51554 | 33.57% |
| Hospice facility | 5364 | 3.49% |
| Nursing home/long term care | 27205 | 17.72% |
| Other | 6632 | 4.32% |
| Place of death unknown | 372 | 0.24% |

**Supplementary Table 2.** Overall Age Adjusted mortality trends in Hypertension-Related Cardiomyopathy in the United States, 1999-2023

| Year | Age Adjusted Mortality Rate | 95% Lower CI | 95% Upper CI |
| --- | --- | --- | --- |
| 1999 | 1.26 | 1.21 | 1.3 |
| 2000 | 2.54 | 2.47 | 2.61 |
| 2001 | 2.6 | 2.53 | 2.67 |
| 2002 | 2.49 | 2.42 | 2.55 |
| 2003 | 2.58 | 2.51 | 2.64 |
| 2004 | 2.54 | 2.48 | 2.61 |
| 2005 | 2.51 | 2.45 | 2.57 |
| 2006 | 2.51 | 2.44 | 2.57 |
| 2007 | 2.46 | 2.4 | 2.52 |
| 2008 | 2.46 | 2.4 | 2.53 |
| 2009 | 2.42 | 2.36 | 2.48 |
| 2010 | 2.43 | 2.37 | 2.5 |
| 2011 | 2.53 | 2.47 | 2.59 |
| 2012 | 2.47 | 2.41 | 2.53 |
| 2013 | 2.09 | 2.03 | 2.14 |
| 2014 | 2.05 | 1.99 | 2.1 |
| 2015 | 2.01 | 1.96 | 2.06 |
| 2016 | 2 | 1.94 | 2.05 |
| 2017 | 2.01 | 1.95 | 2.06 |
| 2018 | 2 | 1.94 | 2.05 |
| 2019 | 1.97 | 1.92 | 2.02 |
| 2020 | 2.25 | 2.2 | 2.31 |
| 2021 | 2.36 | 2.31 | 2.41 |
| 2022 | 2.34 | 2.29 | 2.4 |
| 2023 | 2.22 | 2.16 | 2.27 |

**Supplementary Table 3.** Overall Trend in Age-Adjusted Mortality Rates for Hypertension-Related mortality trends in Cardiomyopathy by Sex in the United States, 1999-2023

| Year | Male | Female |
| --- | --- | --- |
| 1999 | 1.55 | 1 |
| 2000 | 3.1 | 2.1 |
| 2001 | 3.24 | 2.1 |
| 2002 | 3.19 | 1.99 |
| 2003 | 3.16 | 2.12 |
| 2004 | 3.23 | 2.01 |
| 2005 | 3.21 | 1.94 |
| 2006 | 3.18 | 2.02 |
| 2007 | 3.13 | 1.91 |
| 2008 | 3.17 | 1.93 |
| 2009 | 3.07 | 1.89 |
| 2010 | 3.22 | 1.82 |
| 2011 | 3.31 | 1.93 |
| 2012 | 3.33 | 1.8 |
| 2013 | 2.78 | 1.5 |
| 2014 | 2.82 | 1.42 |
| 2015 | 2.64 | 1.48 |
| 2016 | 2.67 | 1.46 |
| 2017 | 2.67 | 1.48 |
| 2018 | 2.69 | 1.4 |
| 2019 | 2.67 | 1.41 |
| 2020 | 3.02 | 1.58 |
| 2021 | 3.2 | 1.71 |
| 2022 | 3.16 | 1.67 |
| 2023 | 2.98 | 1.61 |

**Supplementary Table 4.** Overall Trend in Age-Adjusted Mortality Rates for Hypertension-Related mortality trends in Cardiomyopathy by States in the United States, 1999-2023

| State | Age-Adjusted Mortality Rate | 95% Lower Confidence Interval | 95% Upper Confidence Interval |
| --- | --- | --- | --- |
| Alabama | 3.03 | 2.76 | 3.3 |
| Alaska | 3.73 | 2.79 | 4.89 |
| Arizona | 4.02 | 3.75 | 4.3 |
| Arkansas | 3.36 | 2.98 | 3.75 |
| California | 5.07 | 4.94 | 5.21 |
| Colorado | 2.95 | 2.67 | 3.25 |
| Connecticut | 2.7 | 2.42 | 3 |
| Delaware | 8.38 | 7.33 | 9.44 |
| District of Columbia | 6.84 | 5.66 | 8.2 |
| Florida | 5.14 | 4.98 | 5.3 |
| Georgia | 4.3 | 4.05 | 4.54 |
| Hawaii | 7.95 | 7.14 | 8.76 |
| Idaho | 3.78 | 3.22 | 4.35 |
| Illinois | 3.47 | 3.29 | 3.66 |
| Indiana | 5 | 4.68 | 5.32 |
| Iowa | 5.16 | 4.7 | 5.63 |
| Kansas | 3.42 | 3.02 | 3.83 |
| Kentucky | 3.74 | 3.41 | 4.08 |
| Louisiana | 4.59 | 4.22 | 4.96 |
| Maine | 4.05 | 3.47 | 4.64 |
| Maryland | 7.38 | 6.96 | 7.78 |
| Massachusetts | 2.08 | 1.89 | 2.27 |
| Michigan | 4.55 | 4.31 | 4.8 |
| Minnesota | 6 | 5.61 | 6.38 |
| Mississippi | 6.42 | 5.87 | 6.96 |
| Missouri | 3.82 | 3.55 | 4.1 |
| Montana | 3.08 | 2.51 | 3.74 |
| Nebraska | 5.5 | 4.86 | 6.13 |
| Nevada | 4.19 | 3.71 | 4.67 |
| New Hampshire | 3.76 | 3.21 | 4.37 |
| New Jersey | 4.35 | 4.11 | 4.59 |
| New Mexico | 2.35 | 1.98 | 2.76 |
| New York | 3.09 | 2.95 | 3.23 |
| North Carolina | 5.6 | 5.33 | 5.87 |
| North Dakota | 4.16 | 3.32 | 5.15 |
| Ohio | 6.26 | 6 | 6.52 |
| Oklahoma | 4.79 | 4.37 | 5.2 |
| Oregon | 4.72 | 4.32 | 5.11 |
| Pennsylvania | 4.97 | 4.76 | 5.19 |
| Rhode Island | 4.2 | 3.52 | 4.97 |
| South Carolina | 6.76 | 6.33 | 7.2 |
| South Dakota | 4.35 | 3.56 | 5.26 |
| Tennessee | 4.93 | 4.62 | 5.24 |
| Texas | 3.98 | 3.83 | 4.13 |
| Utah | 2.2 | 1.84 | 2.61 |
| Vermont | 4.98 | 4.08 | 6.02 |
| Virginia | 4.75 | 4.47 | 5.02 |
| Washington | 4.56 | 4.26 | 4.86 |
| West Virginia | 4.82 | 4.26 | 5.37 |
| Wisconsin | 5.48 | 5.13 | 5.84 |
| Wyoming | 3.95 | 2.98 | 5.09 |

**Supplementary Table 5.** Age-Adjusted Mortality Rates for Hypertension-Related mortality trends in Cardiomyopathy by Census Region in the United States, 1999-2023

| Census Region | Age-Adjusted Mortality Rate (AAMR) | AAMR Lower 95% confidence interval - Upper 95% confidence interval |
| --- | --- | --- |
| Northeast | 3.66 | 3.57-3.74 |
| Midwest | 4.84 | 4.75-4.94 |
| South | 4.86 | 4.79-4.93 |
| West | 4.54 | 4.45-4.63 |

**Supplementary Table 6.** Overall Trend in Age-adjusted Mortality Rates for Hypertension-Related mortality trends in Cardiomyopathy by Urbanization and Race in the United States, 1999-2023

| Year | Metropolitan | Non-metropolitan | American Indian or Alaska Native | Asian or Pacific Islander | Black or African-American | White | Hispanic or Latino |
| --- | --- | --- | --- | --- | --- | --- | --- |
| 1999 | 5.19 | 1.92 |  | 1.16 | 3.17 | 1.05 | 1.29 |
| 2000 | 10.37 | 4.49 |  | 2.67 | 6.7 | 2.13 | 2.2 |
| 2001 | 10.59 | 4.86 |  | 2.28 | 6.91 | 2.16 | 2.17 |
| 2002 | 10.2 | 4.56 | 2.18 | 2.41 | 6.06 | 2.12 | 2.12 |
| 2003 | 10.3 | 4.94 | 2.11 | 2.1 | 6.29 | 2.19 | 2.41 |
| 2004 | 10.26 | 4.89 | 2.73 | 1.64 | 5.97 | 2.18 | 2.5 |
| 2005 | 10.03 | 4.89 | 1.82 | 1.76 | 5.95 | 2.14 | 2.12 |
| 2006 | 10.22 | 4.72 | 1.77 | 2.11 | 5.77 | 2.18 | 2.23 |
| 2007 | 9.85 | 4.95 | 2.56 | 1.68 | 5.34 | 2.16 | 2.22 |
| 2008 | 10.12 | 4.67 | 1.41 | 1.51 | 5.66 | 2.15 | 1.93 |
| 2009 | 9.8 | 4.42 | 2.06 | 1.5 | 5.35 | 2.11 | 2.33 |
| 2010 | 9.79 | 5.01 | 1.37 | 1.48 | 5.38 | 2.15 | 2.17 |
| 2011 | 10.18 | 5.04 | 2.16 | 1.71 | 5.53 | 2.18 | 2.4 |
| 2012 | 9.85 | 4.96 | 1.71 | 1.75 | 5.4 | 2.15 | 2.17 |
| 2013 | 8.4 | 4.02 | 1.46 | 1.42 | 4.23 | 1.84 | 1.89 |
| 2014 | 8.22 | 4.09 | 2.02 | 1.14 | 3.91 | 1.85 | 1.67 |
| 2015 | 7.91 | 4.29 | 2.03 | 1.19 | 3.76 | 1.81 | 1.53 |
| 2016 | 7.91 | 4.11 | 1.09 | 1.3 | 3.65 | 1.83 | 1.51 |
| 2017 | 8.11 | 4.02 | 1.82 | 1.13 | 3.63 | 1.83 | 1.52 |
| 2018 | 8.04 | 4.06 | 1.65 | 1.15 | 3.65 | 1.82 | 1.53 |
| 2019 | 7.91 | 4.24 | 1.36 | 1.02 | 3.64 | 1.79 | 1.52 |
| 2020 | 8.94 | 5.02 | 2.4 | 1.15 | 4.19 | 2.01 | 1.68 |
| 2021 |  |  | 2.47 | 1.04 | 4.3 | 2.21 | 1.67 |
| 2022 |  |  | 1.99 | 1.06 | 4.12 | 2.18 | 1.5 |
| 2023 |  |  | 1.57 | 0.84 | 3.73 | 2.11 | 1.49 |

**Supplementary Table 7.** Crude Mortality Rates for Hypertension-Related mortality trends in Cardiomyopathy by Age-group in the United States, 1999-2023

| Ten-Year Age Groups | Crude Death Rate | 95% Confidence Interval |
| --- | --- | --- |
| Young population | | |
| 25-34 years | 2.42 | 1.76- 3.29 |
| 35-44 years | 9.09 | 7.67 – 10.54 |
| 45-54 years | 24.48 | 22.16-26.9 |
| 55-64 years | 57.36 | 53.38 – 61.32 |
| Older population | | |
| 65-74 years | 124.29 | 117.15 – 131.42 |
| 75-84 years | 298.81 | 284.46 – 313.15 |
| 85+ years | 840.6 | 802.14 – 879.07 |
